# Integrating Multiple Modalities to Enhance Performance for Brain Age Prediction

**DOI:** 10.1002/alz70856_099869

**Published:** 2025-12-24

**Authors:** Fan Zhang, Melissa Petersen, Leigh A. Johnson, James Hall, Sid E. O'Bryant

**Affiliations:** ^1^ Institute for Translational Research, University of North Texas Health Science Center, Fort Worth, TX, USA

## Abstract

**Background:**

Brain age prediction is a valuable tool for identifying deviations from normal aging and detecting neurological disorders such as Alzheimer's disease (AD) at an early stage. With the advent of multimodal databases like HABS‐HD, leveraging multilevel and multiscale AD datasets may enhance the precision of brain age prediction. This study aimed to investigate whether combining serum and plasma biomarkers with feature selection could improve brain age prediction.

**Methods:**

The study included 150 normal controls for serum testing and 100 normal controls for plasma testing, with 65 overlapping normal participants. A 10‐times repeated 5‐fold cross‐validation model was employed to compare performance and reduce overfitting. Feature selection methods were applied to enhance prediction performance by combining serum and plasma biomarkers in brain age prediction.

**Results:**

Predictive performance was evaluated using Root Mean Square Error (RMSE) and the coefficient of determination (R^2^). The “Serum only” model achieved an RMSE of 5.07 and an R^2^ of 0.746, indicating moderate accuracy. The “Plasma only” model improved performance with an RMSE of 4.48 and an R^2^ of 0.786. Combining “Serum + Plasma” further enhanced results, achieving an RMSE of 4.12 and an R^2^ of 0.816, underscoring the synergistic benefits of multimodal integration. The highest performance was observed with “Serum + Plasma + Feature Elimination,” which achieved the lowest RMSE of 2.77 and the highest R^2^ of 0.917, indicating superior predictive accuracy and model fit.

**Conclusion:**

Leveraging machine learning (ML) techniques with comprehensive AD datasets holds significant potential to improve brain age prediction.

